# Sequential versus Simultaneous Quantitative Analysis of Biomarkers in Individual Cells by ICP-MS and Mass Cytometry: A Focus on Immunotherapy

**DOI:** 10.1021/acs.analchem.5c03660

**Published:** 2025-11-12

**Authors:** Ángela de la Rosa-Díaz, Christian Sordo-Bahamonde, Segundo Gonzalez, Mario Corte-Rodriguez, María Montes-Bayón

**Affiliations:** † Department of Physical and Analytical Chemistry, Faculty of Chemistry, 16763 University of Oviedo, c/Julián Clavería 8, Oviedo 33006, Spain; ‡ Health Research Institute of Asturias (ISPA), Avda de Roma s/n, Oviedo 33011, Spain; § Department of Functional Biology, Faculty of Biology, University of Oviedo, c/Julián Clavería s/n, Oviedo 33006, Spain; ∥ Instituto Universitario de Oncología Del Principado de Asturias (IUOPA), Oviedo 33006, Spain

## Abstract

Quantification strategies to establish the content of different elements in individual cells are crucial for elucidating any kind of biological conclusion. Technically, this process is hampered when using inductively coupled plasma mass spectrometry (ICP-MS) with nonsimultaneous mass analyzers. This work aims to establish under which set of conditions the sequential and simultaneous detection of different elements in individual cells provides comparable results. Thus, endogenous elements (P and Fe) and Ir as an exogenous cell marker are sequentially measured and quantified using ICP-MS revealing that cell storage and dilution conditions are more critical than cell deposition within the sample introduction device. Under optimized conditions, the interaction of an immunotherapeutic agent (Rituximab) that directs against the B-lymphocyte antigen called cluster of differentiation 20 (CD20) on a B-cell chronic lymphocytic leukemia cell line (MEC-1) is quantified using both single-cell analysis by ICP-MS (SC-ICP-MS) and mass cytometry (CyTOF) after labeling with Lu. Although the obtained values are in good agreement, a general trend is observed with a slight bias toward higher results in SC-ICP-MS versus CyTOF probably ascribed to the automated data treatment system used in CyTOF, discarding double cell events. Both analytical approaches (SC-ICP-MS and CyTOF) showed comparable levels of CD20 expression around 1 × 10^4^ receptors/cell in good agreement with the literature, proving the suitability of a similar calibration strategy for both instruments. The application of CyTOF quantification strategies has permitted the analysis of CD20 in healthy subjects and patient samples as proof of concept for the applicability of the proposed work.

## Graphical abstract

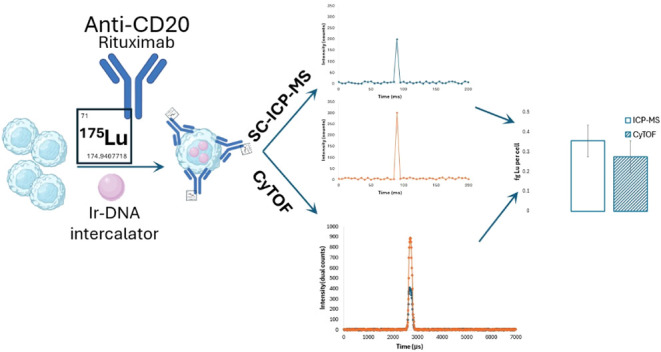

## Introduction

Immunotherapies represent a broad and rapidly growing group of therapies having a substantial impact on cancer outcomes.^1^ Their strength relies on their potential to activate the immune system to specifically target cancer cells without the broadly damaging side effects of many conventional chemotherapeutics. Monoclonal antibodies (mAbs) were among the initial types of immunotherapy approved as anticancer treatments and continue to play a pivotal and growing role in current treatment regimens.^2^ Rituximab (IDEC Pharmaceuticals, La Jolla, CA, USA), a monoclonal antibody against the cluster of differentiation 20 (CD20) antigen, was one of the first FDA-approved immunotherapeutic agents (1997) that was shown to be an effective single-agent treatment for B-cell non-Hodgkin lymphomas.^3,4^ Structurally, this is an anti-CD20 chimeric antibody with human IgG1 immunoglobulin constant regions and variable regions from an anti-CD20 murine antibody.^5^ CD20 is a transmembrane protein marker expressed on the surface of B-cells, as well as many B-cell malignancies, and is also believed to function as a calcium ion channel. Mechanistically, once rituximab binds to CD20-positive cells, cell death is induced by various mechanisms, including antibody-dependent cell-mediated cytotoxicity, antibody-dependent phagocytosis, complement-derived cytotoxicity, and the direct apoptotic effect of rituximab binding to CD20.^6^

However, despite its widespread use, there is still much uncertainty regarding the mechanism(s) of action of Rituximab *in vivo* and there is also a lack of effective predictive biomarkers to identify which patients will respond to this treatment.^7^ In this regard, several studies have suggested that the level of cell surface antigen expression (e.g., CD20) may affect the response to monoclonal antibody-based therapy.^8^ Thus, the development of bioanalytical methods that permit us to establish, quantitatively, the effectiveness of Rituximab binding to CD20 in patient cancer cells could help predict the therapeutic outcome of the drug before patient administration. As the concept of “personalized medicine” gains popularity, it is highly desirable to discover a prognostic indicator of response to this type of therapy and, more specifically, to identify the antibody-based regimen that is likely to be most effective in an individual patient.

With the aim to determine the relationship between levels of antigen expression and the response to monoclonal antibody therapy, the level of surface CD20 (and the clusters of differentiation 22, 25, and 52, named CD22, CD25, and CD52, respectively) expression has been previously estimated using flow cytometry.^9^ In that study, the antibody binding capacity (ABC) was calculated using fluorescent beads to normalize fluorescent intensities obtained in individual cells after using saturating concentrations of the labeled antibody. However, the increasing use of mass cytometry as an alternative to flow cytometry for multiplex detection of several (up to 70) biomarkers per cell and the increasing demand for absolute values rather than comparative levels of expression of specific biomarkers in individual cells require the development of quantitative and absolute methods of analysis.^10,11^ At this point, the previously developed quantitative experiments of single-cell inductively coupled plasma mass spectrometry (SC-ICP-MS) can be of extraordinary importance.^12^ Among various mass spectrometric techniques, SC-ICP-MS has been proven to be a versatile tool for studies on the determination of constitutive elements,^13,14^ the cellular uptake of metal-containing drugs,^15^ and the incorporation of metallic nanoparticles in individual cells.^16,17^ In combination with labeling strategies using metal-containing probes (antibodies), the quantification of specific protein biomarkers has been conducted using monoplexed methodologies.^18,19^ This is mostly due to the limitation of most ICP-MS mass analyzers (quadrupoles) for monitoring more than one isotope in fast (microsecond-range), transient, multi-isotopic signals originated by individual cells. The inclusion into the market of the CyTOF instrument with the capabilities of conducting such kind of measurements has opened many possibilities for quantitative experiments.^20,21^ However, adequate quantitative strategies must be developed and tested before being routinely used.

In this work, two quantification methodologies will be applied and compared using SC-ICP-MS and CyTOF in the specific problem of addressing the absolute number of CD20 receptors present in different cell suspensions. In both the case of SC-ICP-MS and CyTOF, the use of calibration strategies is based on the application of inorganic standards but taking into account the singularities of CyTOF. A comparison of both techniques, as well as the effect of sequential versus simultaneous monitoring of cell elemental markers, will also be evaluated. The quantification possibilities will be applied to cell cultures of chronic lymphocytic leukemia (MEC-1) and to samples of isolated lymphocytes from healthy individuals and patients with chronic lymphocytic leukemia.

## Materials and Methods

### Instrumentation

All ICP-MS experiments during this study were performed using the triple quadrupole instrument iCAP TQ-ICP-MS (Thermo Fisher Scientific, Bremen, Germany) in the oxygen-TQ mode for the measurement of phosphorus (mass shift from ^31^P^+^ to ^31^P^16^O^+^ after reaction with oxygen in the reaction cell), an on-mass approach using helium as a collision gas to break down the ^40^Ar^16^O^+^ interference on the most abundant isotope of iron ^56^Fe^+^ and SQ-mode (single quadrupole mode) for ^175^Lu^+^ and ^193^Ir^+^ monitoring. For the single-cell experiments, the ICP-MS instrument was fitted with the High Efficiency Sample Introduction System (HE-SIS, Glass Expansion, Weilburg, Germany). The cells were pumped using a microflow syringe pump SP101i (Florida, USA) fitted with a 1 mL Hamilton syringe (Nevada, USA) at 10 μL min^–1^. The data were recorded in time-resolved analysis mode during 3 min per analysis using a dwell time of 5 ms. Under these conditions, only a single isotope could be measured in one run due to the sequential nature of the measurements in a quadrupole system. The studies of the labeled antibody were carried out by connecting online the size exclusion chromatography (SEC), using an HPLC system Agilent 1260 equipped with a quaternary pump (Agilent Technologies, Tokyo, Japan), to the iCAP TQ-ICP-MS instrument as an elemental detector. The column used for the separation was a Superdex 200 10/300 GL (300 × 10 mm, GE Healthcare, Merck, Germany) that has a fractionation range from 10 to 600 kDa. The cell number was determined by absolute counting in a Neubauer chamber.

The CyTOF experiments were conducted in the CyTOF XT Model (Standard BioTools, San Francisco, CA, USA). The CyTOF technology is based on ICP-MS technology including a time-of-flight (TOF) mass analyzer.^22^ In order to maximize sensitivity, the mass range is limited to *m*/*z* = 75–209. The sample introduction system consists of an autosampler using a two-valve combination that handles the sample introduction to the nebulizer and the washing of all tubing and the nebulizer after each sample. Therefore, the sample introduction and cleaning between specimens are fully automatized. The samples are placed in vials in a refrigerated carrousel at 4 °C. Before measurement, each sample is shaken, and EQ6 Calibration Beads (Standard BioTools) are added at a 10% dilution. These beads contain natural isotopic compositions of Y, In, Ce, Tb, Lu, and Bi and are used for instrument calibration and data normalization. Once mixed with the sample, the suspension is loaded into a 300 μL loop and then pumped into the nebulizer at a constant flow rate of 30 μL min^–1^. The microflow concentric nebulizer was placed in a spray chamber heated at 200 °C. This reduced the amount of solvent being introduced into the plasma, avoiding a decrease in plasma temperature and ionization efficiency due to solvent effects.

### Cell Samples

B-cell chronic lymphocytic leukemia cell line MEC-1 was grown in Iscove’s Modified Dulbecco’s Medium (IMDM, LabClinics, Barcelona, Spain) supplemented with 10% (v/v) fetal bovine serum (Gibco, Life Technologies, Madrid, Spain). A2780 ovarian cancer cells (used for optimization) were grown in Roswell Park Memorial Institute 1640 Medium (RPMI 1640, Invitrogen, Fisher Scientific, Madrid, Spain) supplemented with 10% (v/v) fetal bovine serum. The culture medium was always additionally supplemented with 5 μg mL^–1^ of plasmocin prophylactic (InvivoGen, Nucliber, Madrid, Spain). Cells were grown in T-25 flasks at 37 °C in a 5% (v/v) CO_2_ atmosphere. Then, the cells were washed with PBS (three times).

KARPAS-299 cells were kindly provided by Dr. E. Rioja and correspond to a T-cell lymphoma cell line established from the peripheral blood of a 25-year-old man with T-cell non-Hodgkin’s lymphoma. The cells were grown in RPMI 1640 supplemented with 10% FCS (or FBS), 2 mM glutamine, and 100 U/mL penicillin/streptomycin. Both MEC-1 and KARPAS-299 are nonadherent cells and grow in suspension; therefore, the flasks have to be positioned vertically.

Healthy donors and patient samples were obtained by the Hospital Universitario Central de Asturias and will be used solely for the purposes approved in the study protocol. Informed consent was obtained prior to sample collection, and all applicable ethical regulations of the Ethics Committee of the University of Oviedo and Hospital Universitario Central de Asturias have been followed.

### Labeling of Rituximab (Anti-CD20) Using MAXPAR Technology

The solution of clinically used Rituximab (10 mg mL^–1^) was kindly provided by the Hospital Universitario Central de Asturias and further labeled using a Maxpar X8 Antibody Labeling Kit (Fluidigm, San Francisco, CA, USA), following the instructions of the manufacturer and using Lu as the elemental marker. For the reduction of the antibody, tris­(2-carboxyethyl) phosphine (TCEP) was purchased from Sigma-Aldrich. For the purification steps, centrifugal filter units of 3 kDa and 50 kDa were used (Amicon Ultra 0.5 mL, Merck Millipore). The concentration of the labeled antibody was measured spectrophotometrically using a NanoDrop 2000 (Thermo Fisher Scientific) at 280 nm.

The efficiency of the labeling and the stoichiometry of the Lu atoms per molecule of antibody were carried out by connecting the size exclusion chromatography (SEC) online to the iCAP TQ ICP-MS instrument as an elemental detector. An aliquot of 50 μL of a water-diluted labeled antibody solution was injected into the column. The chromatographic separation was performed in isocratic mode for 45 min using 50 mM ammonium acetate as the mobile phase at a flow rate of 0.7 mL min^–1^. The concentration of Lu in the eluting peak of the antibody was addressed by flow injection of inorganic Lu standards of known concentration. The stoichiometry was obtained by correlating the antibody and Lu concentrations.

### Cell Fixation and Staining with the Ir Intercalator and with the Anti-CD20 Antibody

In the case of the A2780 cells, they were collected using trypsin as the dissociation reagent; in the case of MEC-1 cells, since they do not grow adherent, they were just collected by centrifugation. The number of cells was determined by using a Neubauer counting chamber. The sample was loaded into the Neubauer chamber, and cells were manually counted under a light microscope. Cell samples for mass cytometric analyses were prepared according to published procedures.^11^ In brief, 1–3 million cells were processed following the Maxpar Cell Surface Staining with Fresh Fix protocol (Standard BioTools). Cells were first resuspended in Maxpar Cell Staining Buffer and incubated with the appropriate metal-conjugated antibody (anti-CD20) for 30 min at room temperature. Following staining, cells were washed twice with Maxpar Cell Staining Buffer to remove unbound antibodies.

For fixation, cells were resuspended in 1 mL of a freshly prepared 1.6% formaldehyde solution and gently vortexed to ensure thorough mixing. The suspension was incubated for 15 min at room temperature.

Following fixation, the intercalation of Ir was carried out by incubating the cells in 1 mL of Maxpar Fix and Perm Buffer containing 125 nM Cell-ID Intercalator-Ir (Standard BioTools) overnight at 4 °C to label cellular DNA. The next day, samples were washed twice with Maxpar Cell Staining Buffer, followed by two washes with Maxpar Cell Acquisition Solution, and then resuspended in an appropriate volume of Cell Acquisition Solution at a concentration of 0.5 million cells per milliliter. The standard CyTOF XT injection protocol includes a step involving the addition of six metal-loaded calibration beads (EQ Four Element Calibration Beads, Standard BioTools Inc., South San Francisco, CA, USA) in a 1:10 dilution.

### Data Acquisition and Processing

The data acquired by SC-ICP-MS were exported as .csv files and processed using an in-house-prepared Microsoft Excel file. The raw data consist of a time-intensity matrix that must be processed in order to extract the events (short transient signal pulses produced by a cell or a group of cells entering the plasma at the same time) from the background. Since the dwell time in this instrument was set to 5 ms, each cell produces an event with only one data point. The threshold to consider a data point as an event is set with an iterative process that extracts all events higher than the mean of all data points plus 3σ (being σ the standard deviation of the whole data set). This process is repeated until no new events are found. Once all events are collected in an event data set, they are processed to obtain the mass per cell as indicated in the Quantification section.

CyTOF data acquisition takes place with a dwell time of 13 μs. A cell event is comprised of several data points (between 10 and 150). The units for signal intensity are called Dual Counts (DC), which refer to the number of ions being detected in the two measurement modes: pulse counting (digital) and integrated intensity (analogue). For practical purposes, DC is equivalent to counts in other ICP-MS instruments. The initial event filtration is done internally by the integrated software of CyTOF. It uses the selected elements fixed on the acquisition template to identify the corresponding cell events when the signal for those exceeds a threshold of 10 DC. After event detection, the ion counts for every event are summed, and the integrated intensities are set in a flow cytometry standard FCS 3.0 file that can be processed using any flow cytometry software. In this case, FlowJo (Tree Star, Ashland, OR) was used.

CyTOF data postprocessing includes a gating sequence that removes EQ6 Calibration Beads data and cell multiplets or artifacts from the data set.^23,24^ This is based on the duration, shape, and symmetry of the events and the metal content of the beads, which makes them clearly distinguishable from the cells (containing only Ir and Lu as the labeling metals). An example of a typical gating sequence is shown in Figure S1.

### Quantification

Quantification strategies based on the use of liquid inorganic standards by constructing calibration curves for each of the elements under evaluation (P, Ir, Fe, and Lu in the case of SC-ICP-MS and Lu in the case of CyTOF) were performed. In all cases, the intensity (counts) obtained by ICP-MS or CyTOF for the corresponding element in each of these standards was represented versus its concentration to obtain the calibration curve. The mass of each element in the individual cells was calculated using eq 1:

1 $$m_{c}=\frac{\eta \cdot F\cdot t\cdot I}{b}$$

where *m*_c_ is the mass of the element in the cell, η is the transport efficiency of the inorganic standard solutions, *F* is the sample flow into the system, *t* is the integration time, *I* is the count rate intensity of the corresponding cell event (one point) for SC-ICP-MS, and the sum of the intensity of each point corresponding to an individual cell (between 10 and 150 points) in the case of CyTOF, and *b* is the slope of the calibration curves previously obtained. The transport efficiency of the inorganic standard solutions was calculated using the particle number method^25^ with quality control material of gold nanoparticles LGCQC5050 (LGC, Teddington, UK). Briefly, the number of detected particles is compared with the number of introduced particles, taking into account the sample flow rate, the particle number concentration of the standard, and the measurement time. Being small enough (30 nm), their nebulization and transport efficiency to the plasma are assumed to be the same as that of the inorganic standards. The calculated transport efficiency using the total consumption nebulizer used in this work turned out to be about 48% in the SC-ICP-MS, while in the CyTOF system the transport efficiency, measured in the same way, corresponded to about 15%.

## Results and Discussion

### Optimization of Sequential Measurements of Multiple Elements in Individual Cells by SC-ICP-MS

One of the main limitations of using quadrupoles as mass analyzers for elemental fast transient signal acquisition is the inability to monitor all isotopic signals simultaneously within the same cell event. Alternatively, the sequential monitoring of different elements could be applied to the same cell suspension provided that cells remain stable during the multielemental measurement process. Although such a possibility does not secure the presence of all the elements within the same cell, it provides an alternative for multielemental analysis when TOF mass analyzers are not available or when they do not show enough sensitivity. Therefore, in the first part of this study, we focused on the optimization of the conditions to obtain comparative results for sequential measurements using ICP-TQ-MS and the A2780 well-characterized cell model. Different cell markers were used: two endogenous (phosphorus and iron) and one exogenous (iridium). If labeling with the Ir intercalator is correct, the same number of cell events is expected for all the elements, provided that they exhibit adequate sensitivity in the ICP-TQ-MS. The first measurements were taken using Ir first (on mass) for 2 min and then continuing with P (as PO^+^) for two more minutes. The obtained results revealed a significantly lower number of events (data not shown) for P ascribed to the deposit of cells within the syringe and/or containers. Therefore, a series of experiments were designed in order to address this effect by measuring, continuously, each element for 10 min and collecting the data in 2 min intervals (without refilling the syringe). For every set of measurements of a new element, the syringe was refilled, and the cell suspension was carefully shaken. The measured analytes followed the sequence Ir, P and Fe, and the results are shown in Figure 1 as the number of events obtained for each element in the individual runs. The experiments confirmed the cell deposition within each run for all of the elements, Fe being the less affected one (with variations among runs below 10% in most cases) and Ir the most affected one (variations between 15 and 35%). It is noteworthy that after the fourth run, the number of events for all the elements shows statistically similar results. Since every element was measured after refilling the syringe, these results reveal cell deposition, not only in the syringe but also in the container, where cells were stored before analysis. This would explain that Fe (the last element to be measured) shows a similar event number in all cases.

**Figure 1:**
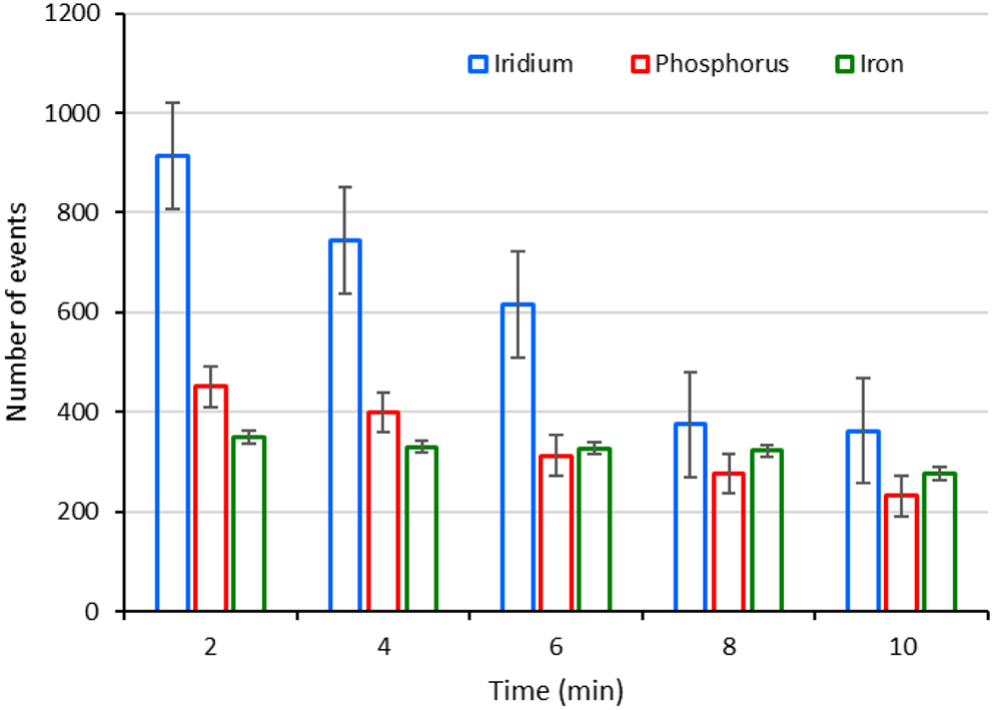
Number of events detected in the cell model A2780 using SC-ICP-MS following the sequence Ir, P, and Fe in 5 individual runs, where each element was measured for 10 min and collecting the data in 2 min intervals (without refilling the syringe). For every set of measurements of a new element, the syringe was refilled and the cell suspension was carefully shaken.

The next step was to evaluate how this cell precipitation affected the quantitative results using the procedure previously described. The results for the different runs (see Figure S2 for iridium (A), iron (B), and phosphorus (C)) revealed a median ranging from 0.4 to 0.6 fg Ir/cell, 7 to 9 fg Fe/cell, and 110 to 220 fg P/cell. In this case, the largest variations were obtained for P while for iron, variations were below 10%. As previously observed in the number of events, after 6 min, the variation of the median is below 20% in all cases. Considering that Fe was the last element to be measured, it could be plausible to believe that cells with the highest content of the element had precipitated in the container and were no longer detectable any longer. Therefore, after 6 min the number of events for the three elements was similar, and the intracellular mass of each of them in the individual cells remained constant. This experiment was repeated by varying the order of measurement of the elements, obtaining similar results.

Besides refilling the syringe, the other parameter to be considered for optimization was the storage conditions before measurement. It is worth mentioning that the experiments of Figure 1 were done by diluting the cell suspension in ultrapure water just before the first measurement and keeping the samples at room temperature throughout the whole experiment. The modification of the conditions involving the dilution of the samples immediately before loading the syringe and the storage of the cell suspension at 4 °C until that moment provided a similar number of events for all the elements in all replicates (see Figure S3) from the beginning. Thus, these conditions were adopted for the rest of the experiments when comparing quantitative SC-ICP-MS and CyTOF.

### Evaluation of the Labeling Process of Rituximab

As previously stated, Rituximab is the commercial name of the anti-CD20 chimeric antibody used to treat B-cell non-Hodgkin lymphomas. According to the formulation, this antibody is stored in a stabilizing solution containing several additives (e.g., polysorbate 80) that could affect the labeling using the Maxpar technology. Therefore, before labeling, the antibody was purified by ultrafiltration using a 50 kDa membrane filter and then labeled using the Lu-containing Maxpar labeling kit. The chromatographic separation of the obtained products can be observed in Figure 2 where two different species can be detected by monitoring the Lu signal, the largest at 15.5 min corresponding to the labeled antibody and the second one at about 20 min corresponding to the unremoved Maxpar excess.

**Figure 2:**
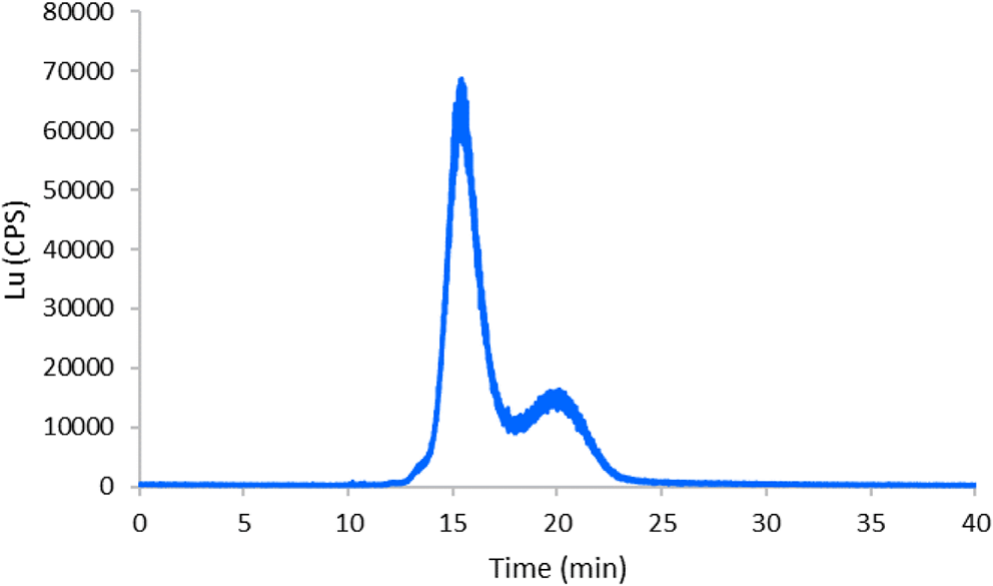
SEC-ICP-MS quantification of Rituximab after purification by ultrafiltration using a 50 kDa membrane filter and then labeling using the Lu-containing Maxpar labeling kit. The species eluting at 15.5 min corresponds to the labeled antibody, and the second peak at about 20 min corresponds to the unremoved Maxpar excess.

These results showed good agreement with previous labeling procedures^18,19^ using commercial pure antibodies rather than a pharmaceutical preparation. The quantification of the antibody after labeling was conducted by spectrophotometry at 280 nm, and the total elemental concentration of Lu was obtained by ICP-MS. Considering a quantitative elution on the SEC-ICP-MS chromatogram, the mass of Lu in the antibody was obtained and thus, the stoichiometric ratio Lu:Ab which turned out to be 76 ± 2. This value agrees with previously obtained stoichiometries (between 30 and 200, depending on the antibody clone) using Maxpar X8 and can be used to translate the Lu mass of individual cells into the number of CD20 receptors per cell.

### Quantitative Strategies: SC-ICP-MS versus CyTOF for Cells Treated with Lu-Labeled Anti-CD20 in MEC-1 Cells

The knowledge of the levels of expression of potential target antigens by the malignant cells, CD20 in this case, could provide a possible rationale for the selection of a specific monoclonal antibody for therapy. With this aim, MEC-1 cultured cells and patient samples of chronic lymphocytic leukemia were treated with the Lu-labeled rituximab according to the procedures section. Two instrumental setups were tested for Lu quantification: SC-ICP-MS and CyTOF. In both cases, apply inorganic Lu standards for calibration. Iridium was used as a cell marker to be measured simultaneously by CyTOF and sequentially under previously optimized conditions by SC-ICP-MS. Antibody titration was conducted to ensure complete labeling of all the receptors (see Figure S4). Briefly, cell samples were incubated with increasing amounts of antibody, until the signal intensity was not increased. The labeling concentration of the antibody was chosen as the minimum amount producing maximum signal intensities (0.5 μg per sample). Figure 3A shows the number of events detected for P (endogenous), Ir (cell marker), and Lu (anti-CD20) for three sequential measurements of the MEC-1 cells by SC-ICP-MS. As can be seen, under the previously optimized conditions, no significant differences can be detected between the three elements using a Student’s *t* test (5% confidence), except for the Ir in replicate 2. Even in this case, phosphorus and lutetium showed a comparable number of events. Therefore, Lu quantification was further done by applying eq 1 with calculated transport efficiencies based on the analysis of gold nanoparticles.

**Figure 3:**
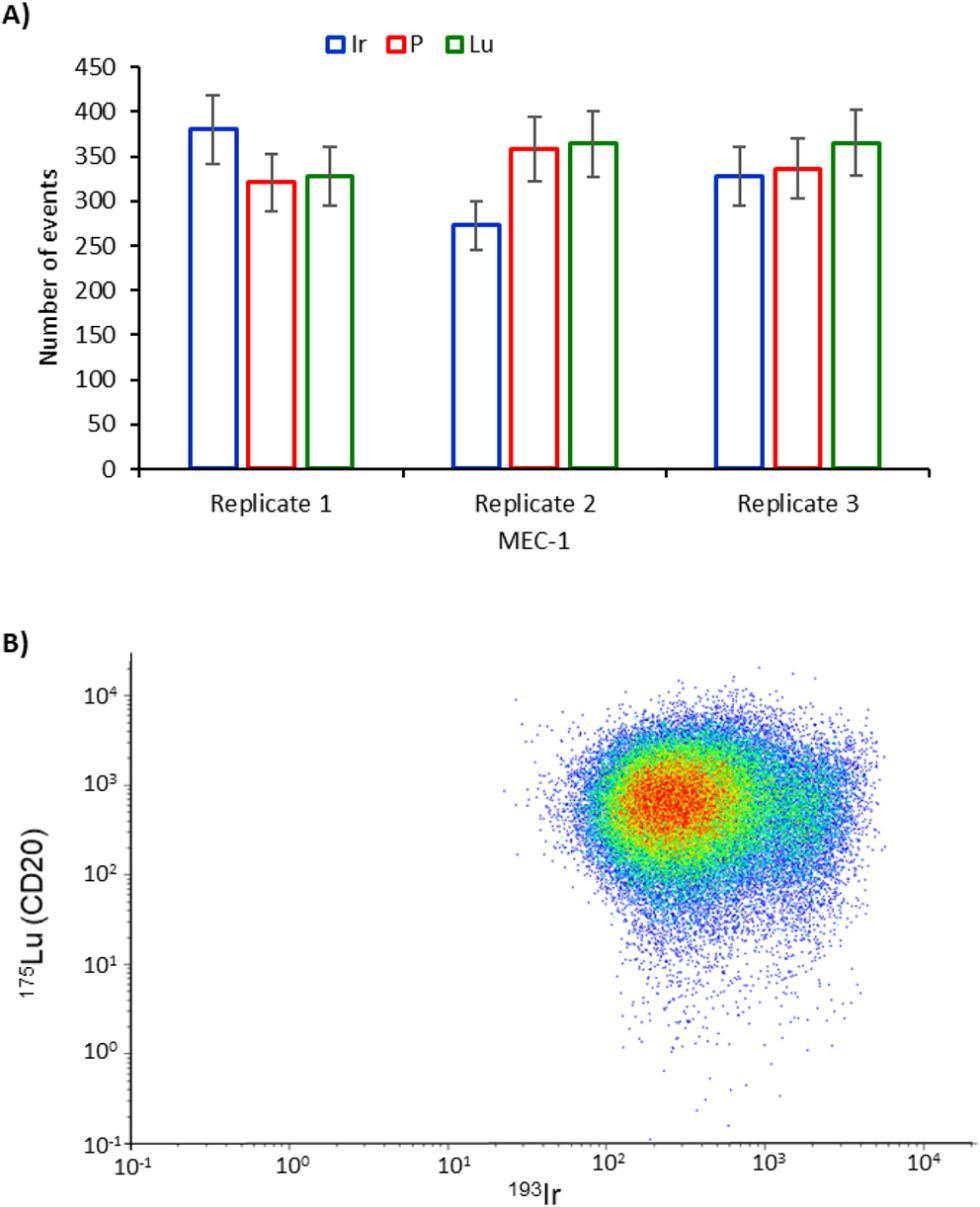
A) The number of events detected for the MEC-1 cells after labeling with Rituximab and monitoring Ir, P, and Lu by SC-ICP-MS. B) Same cell suspension analyzed by CyTOF, measuring Lu and Ir simultaneously within the same cell event.

Similarly, the same samples were then analyzed using CyTOF. For this aim, the standards were acquired, and after adequate calculations, the calibration curve correlating event intensity with the mass of Lu was constructed. It is important to note that, whereas the standard solution contains natural isotopic compositions of Lu, the antibodies were labeled with isotopically enriched ^175^Lu. Therefore, adequate corrections were made to consider the difference in the isotopic composition of natural Lu used in the standards applied for calibration (97.4% ^175^Lu) and the isotopically pure ^175^Lu used for the labeling. The dot-plot diagram of the cell event intensity observed in the CyTOF is shown in Figure 3B for the MEC-1 cells representing the intact cell events containing Ir and Lu (about 3.6 × 10^4^ measured cells) and revealing a higher cell density (red dots) in a relatively homogeneous distribution.

Quantitative results using the two strategies for the MEC-1 cells are shown in Figure 4 for four independent cell cultures analyzed in parallel using SC-ICP-MS and CyTOF and measured on different days. The SC-ICP-MS results revealed a Lu content of 0.18 fg Lu/cell, calculated as the mean of medians obtained for each replicate. In all cases, the number of measured cell events was around 300. On the other hand, CyTOF yielded a comparable result, with a mean of the medians of 0.17 fg Lu/cell (about 36,000 cell events). Since these are not Gaussian distributions, using the mean of the medians represents a more robust indication of the central tendency of the data. A Student’s *t* test to compare these values indicated no significant differences between the SC-ICP-MS and the CyTOF data. However, a general trend observed for the quantitative results revealed a slight bias toward higher values in SC-ICP-MS versus CyTOF, which led to significant differences when comparing the complete distributions according to the Mann–Whitney *U* test (*p* < 0.05). This could be ascribed to the automated procedure of data treatment by which multiple-cell events are discarded in CyTOF analysis during data cleanup^23^ (manually done in the case of SC-ICP-MS). This reason also explains the broader dispersion of the data from SC-ICP-MS. Since cleaning out multiple-cell events is more efficient in CyTOF, a part of the cell distribution is removed, which reduces data dispersion. However, if the Mann–Whitney *U* test is applied to the full data set of replicates after outlier removal, differences are again not significant. Therefore, both systems can be applied to accurately quantify the interaction of Rituximab with the CD20 antigen, although the already mentioned limitations must be taken into account in SC-ICP-MS. When translating these values into the number of receptors per cell by considering the stoichiometry of the Lu atoms per antibody, the obtained values are in the range of 1.0 × 10^4^ per cell in the MEC-1 cells. These results are similar to the levels obtained in lymphocytic leukemia patients (about 9–10 × 10^3^) reported in a previous publication using flow cytometry.^8^

**Figure 4:**
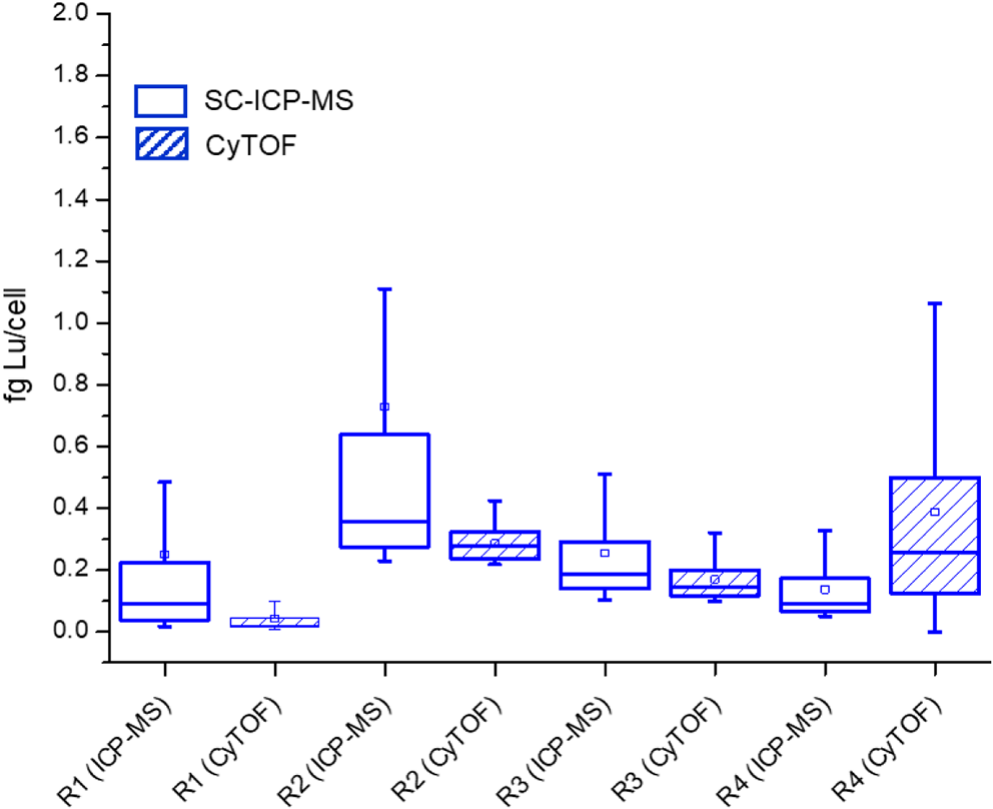
Comparative results obtained for the expression of CD20 in MEC-1 cells using the SC-ICP-MS and CyTOF approaches after labeling with Lu. Empty boxes correspond to SC-ICP-MS data, and dashed boxes correspond to CyTOF results.

Using the same kind of experimental approach and CyTOF, it was possible to quantitatively address the loss of CD20 expression along the life cycle of the MEC-1 cell line. The frequency and mechanisms of target loss are observed during normal plasma cell differentiation and can also occur as a mechanism of resistance to anti-CD20 therapies, such as in B-cell lymphomas, where it is mediated by mechanisms like antigen modulation, gene mutation, or epigenetic changes.^26^ When the number of cell passages exceeds 30 (compared to 12 for Figures 3 and 4), a broader distribution of CD20 expression toward lower values can be observed using CyTOF (see Figure S5A). In this case, 65% of the cells showed a clearly positive expression of CD20 at around 4 × 10^2^–4 × 10^3^ dual counts, while 24% of the cells showed a dimmed expression of CD20, ranging from 10^1^ to 10^2^. The global quantitative CyTOF results showed a median of about 0.06 fg/cell, which agrees well with the SC-ICP-MS (Figure S5B) and is approximately 4–5 times lower than the values reported in Figure 4.

As an approximation, it is possible to estimate the detection limit of the CyTOF methodology using the calibration curve (as three times the standard deviation of the *y*-intercept divided by the slope). Taking into account that a cell event should be formed by at least 10 data points, a limit of detection of 1.3 × 10^–3^ fg Lu is obtained, which translates into about 60 receptors per cell. This limit of detection, however, should be taken with care. Since the cells are detected via the Ir signal given by the DNA-intercalating cell marker, it is actually possible to detect cells with Lu contents below the LOD or even 0.

To further validate the selectivity of the proposed strategies, another cell line (KARPAS 299), which is a T-cell lymphoma cell line but negative in the expression of CD20, was analyzed using the two optimized strategies. In this case, positive Ir events were observed when using both techniques, but no Lu events could be detected in any of the tested instrumental approaches, in particular using SC-ICP-MS confirming the selectivity of the labeling process. However, for the reasons previously mentioned, some Lu DC can be detected in the Ir-containing cell events when using CyTOF (see Figure S6 for the CyTOF measurements).

Finally, two human samples, one from a healthy donor and the other from a CLL patient, were tested using the CyTOF strategy, with the results of Figure 5. As previously discussed, the variable level of CD20 expression even in healthy individuals complicates a clear differentiation between the two sets of samples. However, it is possible to address a higher cell density (75% versus 18% of the total number of cells) with CD20 overexpression in the CLL patient compared to the healthy individual. In addition, the level of CD20 overexpression increases by a factor of 2–3 in the patient cells as seen in the histograms. By transforming these intensities into CD20 per cell, we can set the CD20-positive cells as those expressing more than 360 CD20 molecules (the threshold set by the horizontal axis separating the upper and lower quadrants). Positive cells in the healthy control are centered around 100 DCs (see the red part of the histogram in Figure 5), which corresponds to about 1800 CD20 molecules per cell. On the other hand, in the CLL patient, the CD20-positive cell population, which overexpresses this marker, is centered around 8 × 10^2^ DCs. This can be transformed into 14,300 CD20 molecules per cell (in agreement with what was previously detected in MEC-1 cell models). According to these data, the CD20+ populations can be very well distinguished between the healthy control and the CLL patient. Nonetheless, we must acknowledge the limited extension of this study with only one healthy control and one CLL patient. This limits our conclusions to this specific proof of concept that should be amplified in the future for a much broader population.

**Figure 5:**
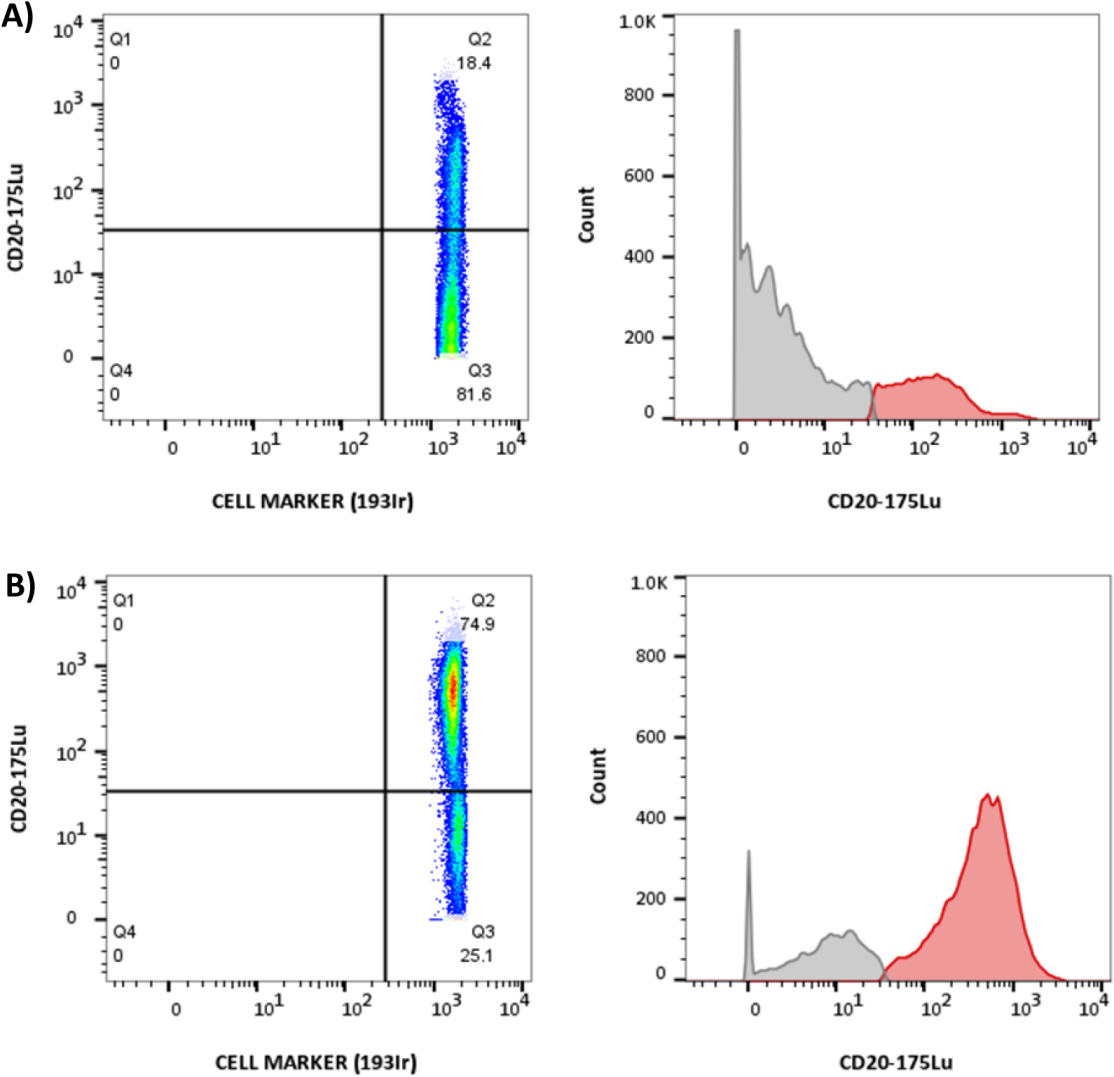
Results obtained by mass cytometry analysis of samples from a healthy control (A) and a CLL patient (B) with the corresponding frequency histograms.

These results all together show the capabilities of both strategies under optimized conditions to provide quantitative results similar to those with adequate sensitivity. The fast acquisition rate of CyTOF permits a higher number of cells to be analyzed, improving the statistics of the data, as well as more specific filtering of double-cell events. This, in combination with adequate data treatment, allows for more discriminative results among cells coming from, in principle, homogeneous cell populations and permits an interesting added value to the obtained results. SC-ICP-MS strategies, however, can still be accurately used as an alternative to CyTOF or other ICP-MS systems with TOF analyzers when these are not available. In the case of using quadrupole-based ICP-MS instruments, however, it must be taken into account the longer analysis times due to the longer dwell times or the lack of access to multielemental single-cell information. Although multiple metals can be analyzed in the same cell suspension, multielemental information in a single cell is not achievable using quadrupole systems because the measurement must be sequential. Additionally, the fast acquisition system of CyTOF allows for the analysis of more concentrated cell samples, which leads to the acquisition of thousands of cells in a shorter time. However, the broader availability of quadrupole systems and their significantly higher sensitivity make them suitable for biomarker expression analysis at the single-cell level.

## Conclusions

The sequential quantification of several elements in a given cell suspension has proven to be adequate when the dilution of the samples is conducted immediately before charging the syringe, and the cell suspensions are stored at 4 °C until that moment. In that case, a similar number of cell events for all of the elements in all of the replicates can be obtained, proving the suitability for sequential quantification. In this regard, the use of inorganic standards applied on ICP-MS for quantification has also shown to be adequate to calculate the elemental content of cells when using CyTOF measurements. When comparing SC-ICP-MS and CyTOF for the same cell suspension labeled with an immunotherapeutic agent, Rituximab, comparable quantitative results are obtained in terms of median values. However, the dispersion of the results is generally higher in the case of SC-ICP-MS where an average number of events of approximately 300 is significantly lower than the 36,000 events measured in CyTOF. There is also a loss of simultaneous single-cell information in SC-ICP-MS due to the sequential detection of different elements, but its high sensitivity may compensate for this drawback for low-expression biomarker quantification. In any case, both approaches have shown promise and may be particularly beneficial for patients with a relatively low expression of the target.

## Supplementary Material



## Notes

Example of a typical gating strategy for CyTOF data cleanup; boxplots obtained for the quantification of iridium, iron, and phosphorus; number of events after optimization of the cells storage conditions; results for the titration of the antibody; obtained results for the analysis of CD20 in degradeted cell samples (Figures S1–S6) (PDF)
