# Supplementary material for: Sequential versus Simultaneous Quantitative Analysis of Biomarkers in Individual Cells by ICP-MS and Mass Cytometry: A Focus on Immunotherapy
Source: Anal Chem. 2025 Nov 12;97(46):25510–8. doi: 10.1021/acs.analchem.5c03660 (PMC12658859; doi:10.1021/acs.analchem.5c03660)
Supplement: Supplementary file 1 [file ac5c03660_si_001.pdf]

SUPPORTING INFORMATION

Sequential versus simultaneous quantitative analysis of biomarkers in individual cells by ICP-MS and mass cytometry: a focus on immunotherapy.

Ángela de la Rosa-Díaz,[1,2] Christian Sordo-Bahamonde,[2,3] Segundo González,[2,3] Mario Corte-Rodriguez[1,2]*, Maria Montes-Bayón.[1,2]*

[1]Department of Physical and Analytical Chemistry. Faculty of Chemistry. University of Oviedo. C/ Julián Clavería 8, 33006, Oviedo, Spain.

[2]Health Research Institute of Asturias (ISPA). Avda de Roma s/n, 33011, Oviedo, Spain

[3]Department of Functional Biology. Faculty of Biology. University of Oviedo. C/ Julián Clavería s/n, 33006, Oviedo, Spain.

*montesmaria@uniovi.es; ORCID: 0000-0001-6114-9405

*cortemario@uniovi.es; ORCID: 0000-0003-0109-4101

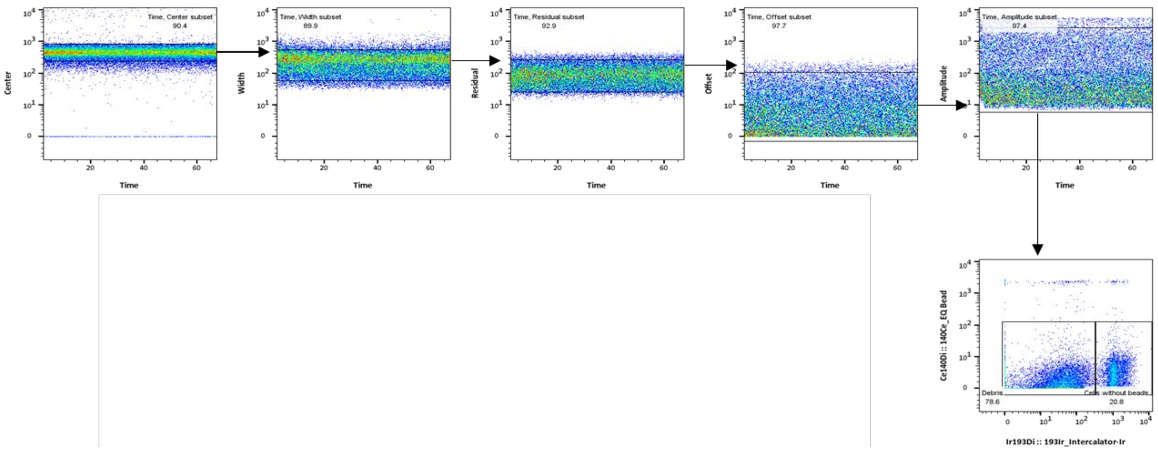

Figure S1. Example of a typical gating strategy for CyTOF data cleanup.

A)

B)

C)

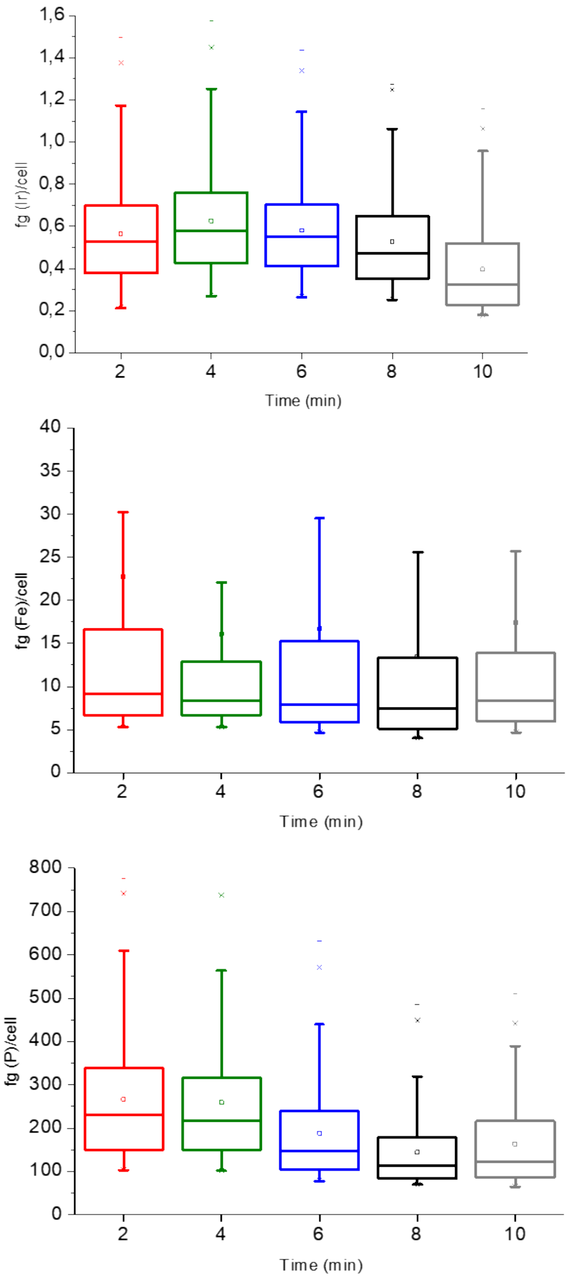

Figure S2. Boxplots obtained for the quantification of iridium (A), iron (B) and phosphorous (C) in A2780 cells with the measurement sequence detailed in the text where each element was measured for 10 min and collecting the data in 2 minutes intervals.

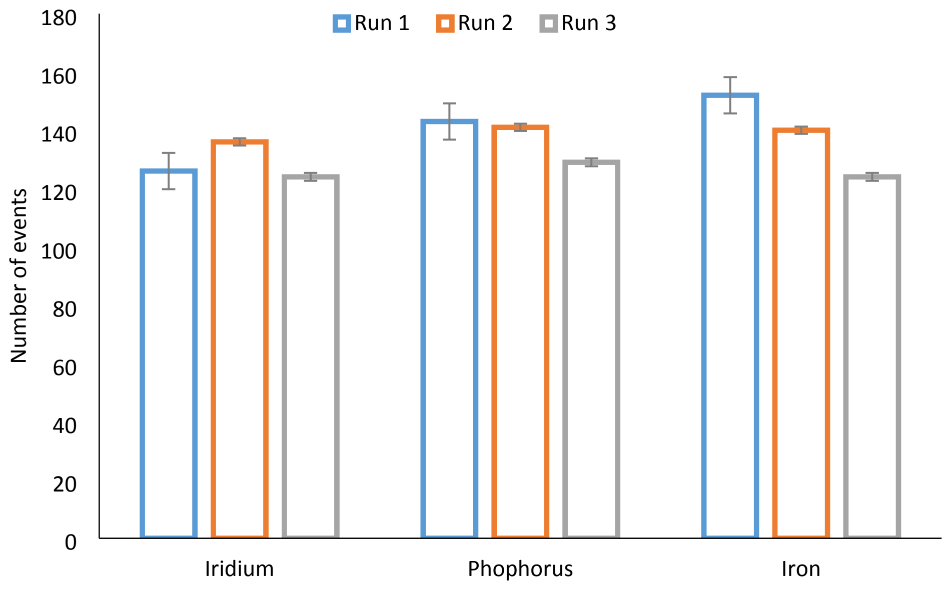

Figure S3. Number of events after optimization of the cells storage conditions.

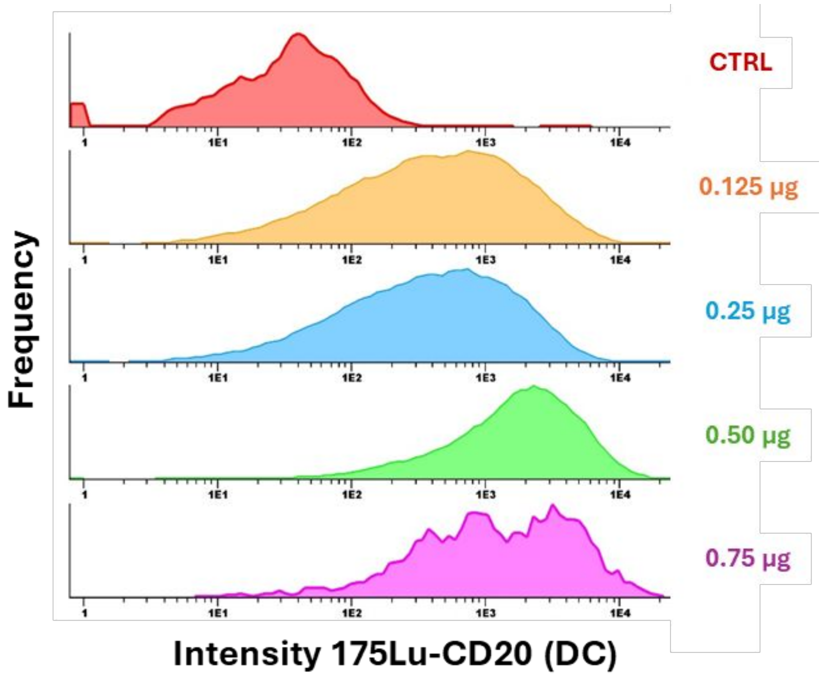

Figure S4. Antibody titration to ensure saturating conditions of CD20 in MEC-1 cells. Antibody mass per incubation on the right panel. X-axis in logarithmic scale.

A)

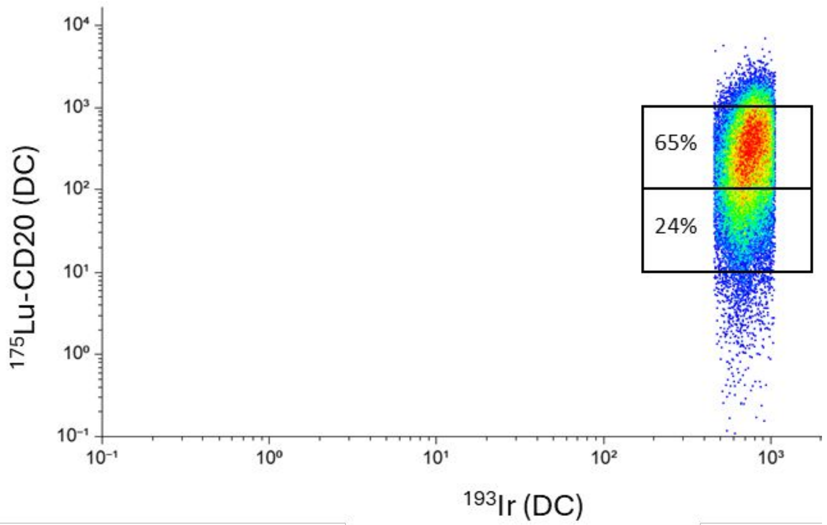

B)

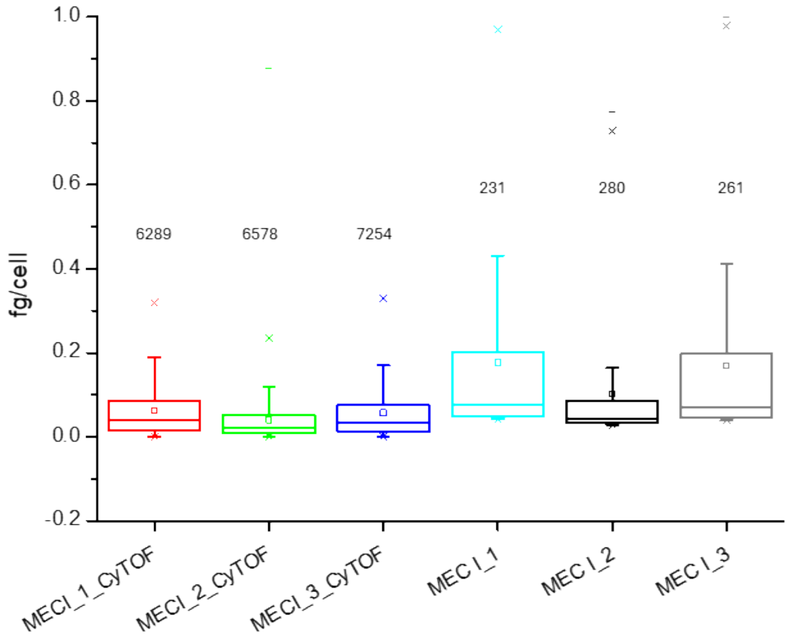

Figure S5. A) Obtained results for the analysis of CD20 as fg Lu/cell in MEC-1 replicates with a number of passes higher than 30. In B), the superscripted numbers correspond to number of events.

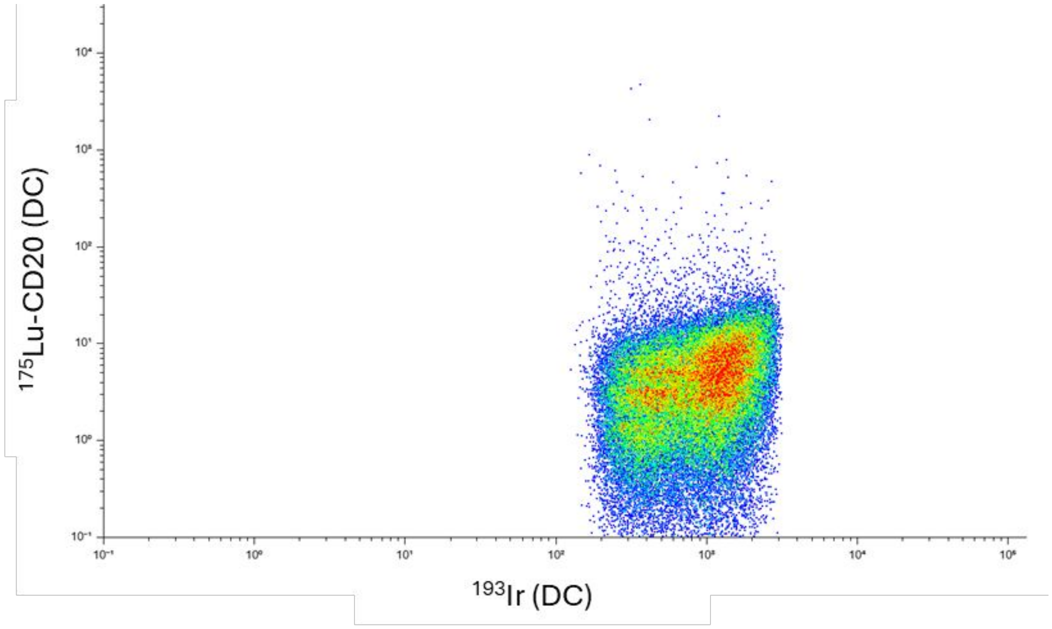

Figure S6. Obtained results for the analysis of CD20 as fg Lu/cell in KARPAS-299.
